# PTBPs: An immunomodulatory-related prognostic biomarker in pan-cancer

**DOI:** 10.3389/fmolb.2022.968458

**Published:** 2022-08-23

**Authors:** Chen Chen, Anquan Shang, Yuting Gao, Jingjuan Huang, Gege Liu, William C. Cho, Dong Li

**Affiliations:** ^1^ Department of Laboratory Medicine, Shanghai Tongji Hospital, School of Medicine, Tongji University, Shanghai, China; ^2^ Department of Clinical Oncology, Queen Elizabeth Hospital, Hong Kong, Hong Kong SAR, China

**Keywords:** biomarkers, immunotherapy, pan-cancer, polypyrimidine tract-binding proteins, prognosis

## Abstract

**Background:** The polypyrimidine tract-binding protein (PTBP) nuclear ribonucleoprotein family of proteins, including PTBP1, PTBP2 and PTBP3, regulate the process of cell proliferation, differentiation, apoptosis and carcinogenesis. PTBPs exhibit oncogenic effects in certain tumors. However, the role of PTBPs in pan-cancer remains unclear. Our study examined the clinical significance and mechanism of PTBPs in pan-cancer.

**Methods:** We compared the expression of *PTBPs* in paired and unpaired tissue samples from the Cancer Genome Atlas (TCGA) database. Univariate and multivariate Cox regression, Kaplan–Meier curves, and time-dependent receiver operating characteristic (ROC) curves were used to assess the prognostic significance of *PTBPs* in pan-cancer. The cBioPortal database also identified genomic abnormalities in *PTBPs*. TISIDB, TCGA, and Cellminer were used to investigate the relationship between *PTBP* expression and immune subtypes, immune checkpoint (ICP) genes, tumor mutational burden (TMB), microsatellite instability (MSI), tumor-infiltrating immune cells, and chemosensitivity. cBioPortal was used to search for *PTBP* co-expressing genes in pan-cancer, and GO and KEGG enrichment analyses were performed to search for *PTBP*-related signaling pathways.

**Results:** *PTBPs* were shown to be widely upregulated in human tumor tissues. *PTBP1* showed good prognostic value in ACC, KIRP, and LGG; *PTBP2* in ACC and KICH; and *PTBP3* in ACC, LGG, and PAAD, with AUC >0.7. *PTBPs* were differentially expressed in tumor immune subtypes and had a strong correlation with tumor-infiltrating lymphocytes (TILs) in the tumor microenvironment (TME). In addition, *PTBP* expressions were related to ICP, TMB, and MSI, suggesting that these three PTBPs may be potential tumor immunotherapeutic targets and predict the efficacy of immunotherapy. Enrichment analysis of co-expressed genes of *PTBPs* showed that they may be involved in alternative splicing, cell cycle, cellular senescence, and protein modification.

**Conclusion:** PTBPs are involved in the malignant progression of tumors. *PTBP1*, *PTBP2* and *PTBP3* may be potential biomarkers for prognosis and immunotherapy in pan-cancer and may be novel immunotherapeutic targets.

## Introduction

Cancer is a life-threatening disease to humans worldwide. The incidence and mortality rates of various cancers have been increasing year by year, and lung cancer, colorectal cancer, liver cancer and gastric cancer have the highest mortality rates (Sung et al., 2021). The field of precision medicine is advancing through new developments in technology and medicine, but the present state of practice is far from ideal (König et al., 2017). Therefore, the identification of tumor-related diagnostic, prognostic, and therapeutic biomarkers is a research hotspot.

Polypyrimidine tract-binding proteins (PTBPs) are important RNA-binding proteins (RBPs) which influence cell growth and development by regulating mRNA stability, translation and alternative splicing (Singh et al., 1995; Mickleburgh et al., 2014). The PTBP family consists of PTBP1, PTBP2 and PTBP3, and these proteins show similarities and differences in expression, structure, and biological function (Oberstrass et al., 2005; Tan et al., 2015). Studies have shown that up-regulation of PTBP1 is associated with the poor prognosis and disease progression in non-muscle-invasive bladder cancer. Therefore, PTBP1 may become a possible outcome-predictor for bladder cancer (Bielli et al., 2018). PTBP2 was also shown to stimulate the proliferation, migration, and metastasis of colorectal cancer cells (Ji et al., 2014). Previous studies demonstrated that PTBP3 enhances the invasion and metastasis of breast cancer and regulates the expression of drug resistance proteins in gastric cancer, suggesting that PTBP3 may serve as a potential novel therapeutic target for gastric cancer (Liang et al., 2017; Hou et al., 2018; Liang et al., 2020). These studies have indicated that PTBPs may function in cancer. However, research has been restricted to a small number of tumor types, and the function of PTBPs in pan-cancer has not been examined.

A growing body of evidence has revealed the close relationship between the tumor microenvironment (TME) and the effectiveness of immunotherapy (Donlon et al., 2021; Newnes et al., 2021). Immune checkpoint (ICP) inhibitors, including PD-1, CTLA4, LAG3, and TIM-3, have potent tumor suppressor effects and can interfere with immune escape; these inhibitors are currently the first-line treatment options for multiple malignancies (Ribas and Wolchok, 2018; Tu et al., 2020). Tumor infiltrating immune cells in TME, such as macrophages, neutrophils, T cells, Treg cells, T helpers, and NK cells, can affect the immunological features of malignancies. Unfortunately, this tumor heterogeneity among individuals influences the efficacy of clinical immunotherapy (Keenan et al., 2019). Thus far, precision medicine has not completely manifested in human tumors. Researchers agree on the need to explore better treatment targets.

In this study, we evaluated the clinical importance and prognostic usefulness of *PTBPs* in pan-cancer. We used the Cancer Genome Atlas (TCGA) to examine the expression levels of *PTBP1*, *PTBP2*, and *PTBP3* in normal and tumor tissues, and cBioPortal was used to examine the genomic alterations. We also examined the link between *PTBP* expression and tumor immune subtype, tumor-infiltrating lymphocytes (TILs), ICP, tumor mutational burden (TMB), microsatellite instability (MSI), and chemosensitivity using multiple databases. Finally, we constructed a PTBP-interacting protein network and performed enrichment analysis of co-expressed genes. Our findings have demonstrated the prognostic value of *PTBPs* in pan-cancer. PTBPs may have excellent potential to be therapeutic targets and predict the efficacy of immunotherapy. We also predicted the molecular mechanisms and biological signaling pathways of *PTBPs* using databases and experimental data.

## Materials and methods

### Study overview

A total of 30 tumor types were studied in this article. A schematic flow chart of our research is shown in Supplementary Figure S1.

### Difference and correlation analysis of *PTBP1*, *PTBP2*, and *PTBP3* expression

Gene expression, clinical data, and survival information for 30 different types of tumors were downloaded from TCGA database (https://portal.gdc.cancer.gov), and the RNA-seq data in level 3 HTSeq-FPKM format were log2-transformed. The Mann–Whitney *U* test was used to analyze the differences in the expression levels of *PTBP1*, *PTBP2*, and *PTBP3* in unpaired tissue samples, and the Wilcoxon signed rank test was used for paired samples.

To analyze the expression correlation between *PTBP1*, *PTBP2*, and *PTBP3* genes, we excluded the tumor types with less than three normal samples and then log2-transformed the ratio of the mean expression of *PTBPs* in tumors and normal samples of the remaining 21 tumor types. All details are shown in Supplementary Table S2.

### Copy number alterations and mutations

Copy number alterations and mutations of *PTBP* genes were analyzed using the online database cBioPortal (http://www.cbioportal.org).

### Cox regression analysis, Kaplan–Meier curve, time-dependent receiver operating characteristic curve and prognostic nomogram

First, univariate Cox regression analysis was performed on the expressions of *PTBP1*, *PTBP2*, and *PTBP3* in pan-cancer. Factors with *p* ≤ 0.05 were included in multivariate Cox regression analysis and displayed in a forest plot. We used Cox regression models to predict survival and plotted Kaplan–Meier (KM) curves. The accuracy of the model in predicting prognosis at a specific time was tested by a time-dependent receiver operating characteristic (ROC) curve, and the prognostic value of *PTBPs* in pan-cancer was determined. Taking the tumor “ACC” as an example, we constructed prognostic nomograms using *PTBP* expressions and pathological stage, and the accuracy of the nomogram was evaluated by a calibration curve. We examined overall survival (OS) in our analyses.

### Analysis of *PTBP* expression and tumor immune subtypes

We analyzed the associations between *PTBP* expression and immune subtypes in human cancers using TISIDB, an online integrated website (http://cis.hku.hk/TISIDB/index.php). *p* ≤ 0.05 was considered statistically significant.

### Relationship between *PTBP* expression and tumor-infiltrating lymphocytes in pan-cancer

Using the ssGSEA algorithm to calculate the score of tumor-infiltrated immune cells in TCGA database, we selected 24 TILs to evaluate the relationship between *PTBP* expressions and tumor-infiltrating lymphocytes (TILs) in pan-cancer. The TILs included activated DCs (aDCs), B cells, CD8 T cells, cytotoxic cells, DC, eosinophils, immature DCs (iDCs), macrophages, mast cells, neutrophils, NK CD56bright cells, NK CD56dim cells, NK cells, plasmacytoid DCs (pDCs), T cells, T helper cells, T central memory (Tcm), T effector memory (Tem), T follicular helper (Tfh), T gamma delta (Tgd), Th1 cells, Th17 cells, Th2 cells, and Tregs (Bindea et al., 2013). Neutrophils and macrophages were selected for detailed display.

### Correlation analysis of *PTBP* expressions with immune checkpoint, tumor mutational burden, and microsatellite instability

Spearman correlation analysis was performed to examine the relation of the expression levels of *PTBPs* and four immune checkpoint (ICP) genes (PD-1, CTLA4, LAG3, and TIM-3 genes). We used Spearman correlation analysis to determine the correlation of *PTBP* expressions with tumor mutational burden (TMB) and MSI in human cancers. RNA-seq data and clinical information were obtained from TCGA database. The TMB data and microsatellite instability (MSI) data were derived from the studies of Thorsson et al. (2018) and Bonneville et al. (2017), respectively. *p* ≤ 0.05 was considered statistically significant.

### Correlation analysis with drug susceptibility

Transcriptome data (RNA: RNA-seq) and drug sensitivity data (compound activity: DTP NCI-60) were downloaded from Cellminer (https://discover.nci.nih.gov/cellminer); these data are derived from the same 60 samples. Only FDA approved samples were analyzed. A positive correlation indicated that the higher the expression of *PTBP*, the more sensitive that the cells were to the drugs.

### Protein-protein interaction network analysis

We performed the analysis of Protein-Protein Interaction (PPI) networks using the STRING website (https://string-db.org/). The parameters for finding the interacting proteins of PTBPs were set as follows: minimum required interaction score [“Medium confidence (0.400)”], meaning of network edges (“evidence”) and active interaction sources (“experiments”). The parameters for finding the interaction relationship among PTBP1, PTBP2 and PTBP3 were set as follows: minimum required interaction score [“Medium confidence (0.400)”], meaning of network edges (“evidence”), active interaction sources (“textmining, experiments, databases, co-expression, co-occurrence”), and max number of interactors to show (“no more than 10 interactors”).

### Co-expressed genes of *PTBPs* in pan-cancer

We downloaded the dataset “Pan-cancer analysis of whole genomes (ICGC/TCGA, Nature 2020)” on cBioPortal and screened several mRNAs co-expressed with *PTBPs* using the absolute value of Spearman’s correlation coefficient ≥0.4 and *p* < 0.05. Data were obtained from 991 samples.

### GO enrichment and KEGG pathway analysis

We performed ID conversion on 926 *PTBP1*-related mRNAs, 657 *PTBP2*-related mRNAs, and 874 *PTBP3*-related mRNAs obtained in the previous step and analyzed their functions by GO and KEGG enrichment analysis. After correcting the *p*-value by the BH method, items with p. adjust ≤0.05 were selected for partial visualization.

### Statistical analysis

R (version 3.6.3) was used for statistical analysis and visualization. The following R packages were used in this study: ggplot2 package [version 3.3.3], ggpubr package [version 0.1.4] for basic drawing, limma package [version 3.28.14] for differential analysis, survminer package [version 0.4.9], survival package [version 3.2–10] for statistical analysis of survival data, timeROC package [version 0.4] for ROC curve analysis, rms package [version 6.2–0] for building nomograms, impute package [version 1.68.0] for processing the missing value, GSVA package [version 1.34.0] for immune infiltration analysis, org. Hs.eg.db package [version 3.10.0] for id conversion, and the clusterProfiler package [version 3.14.3] for enrichment analysis.

## Results

### Expression levels of *PTBPs* in tissues of pan-cancer

**FIGURE 1:**
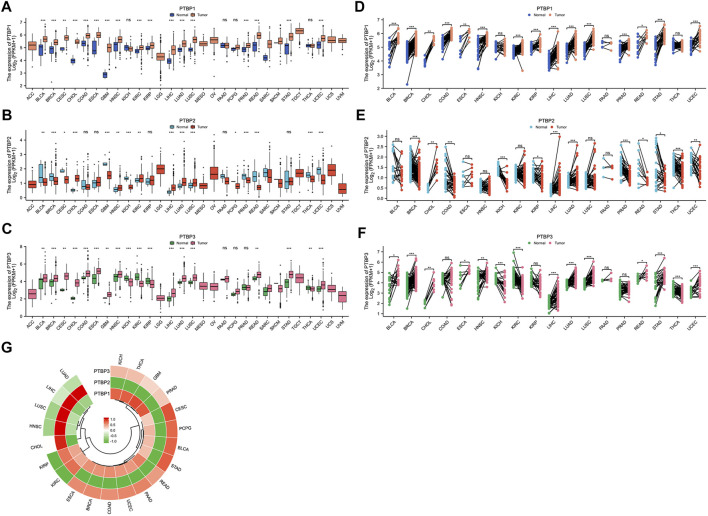
The expression levels of *PTBP* in pan-cancer tissues and the expression correlation among the three *PTBP* genes. RNA-seq data were obtained from the TCGA database. **(A–C)** Differential expression of *PTBPs* in unpaired samples of 30 tumor types. **(D–F)** Differential expression of *PTBPs* in paired samples of 30 tumor types (*p < 0.05; **p < 0.01; ***p < 0.001; ns: not significant). **(G)** Correlation analysis of the expressions of *PTBP1*, *PTBP2*, and *PTBP3* in various tumor types.

We analyzed the expression levels of *PTBPs* in tumor tissues and normal/adjacent tissues from 30 cancer types, and the abbreviations of tumor types are shown in Supplementary Table S1. Paired differential expression analysis was then performed for 18 cancer types with more than three normal samples. Among *PTBPs*, *PTBP1* showed the highest RNA expression level in tumor tissues, followed by *PTBP3*; the expression level of *PTBP2* was the lowest among the three genes. *PTBP1* was upregulated in tumor tissues compared with normal tissues in BLCA, BRCA, CESC, CHOL, COAD, ESCA, GBM, HNSC, KIRC, KIRP, LIHC, LUAD, LUSC, PRAD, READ, STAD, and UCEC. *PTBP2* was upregulated in CHOL, HNSC, KIRC, LIHC, LUAD, and LUSC and downregulated in BLCA, BRCA, CESC, GBM, KICH, PCPG, PRAD, READ, THCA, and UCEC. *PTBP3* was upregulated in BLCA, BRCA, CESC, CHOL, COAD, ESCA, GBM, HNSC, LIHC, LUAD, LUSC, READ, STAD, and UCEC and downregulated in KICH, KIRC, KIRP, and THCA (Figures 1A–C). The results of differential expression analysis for paired samples are shown in Figures 1D–F. Furthermore, we predicted the structures of these three proteins based on AlphaFold (Jumper et al., 2021; Varadi et al., 2022) (Supplementary Figures S2–S4), among which, PTBP1 and PTBP2 were highly similar in structure.

### Expression correlation among *PTBP1*, *PTBP2,* and *PTBP3*

Expression correlation analysis was performed on 21 cancer types with more than three normal samples. As shown in the circular heatmap in Figure 1G, *PTBP1* and *PTBP3* are expressed similarly, while *PTBP2* and *PTBP1/3* have the opposite expression in multiple cancers.

### Genetic alterations of *PTBPs*

**FIGURE 2:**
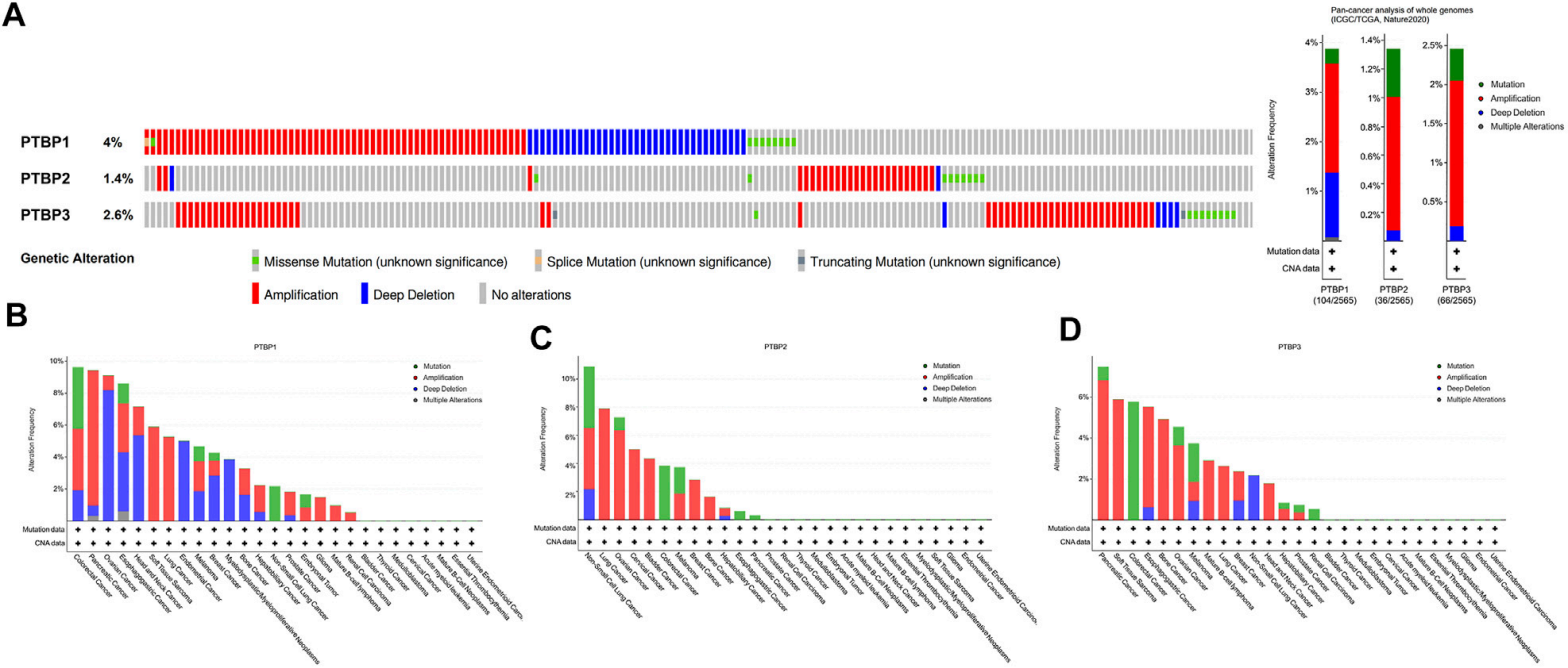
Characterization of genetic alterations in *PTBPs*. **(A)** General profile of genetic alterations in *PTBPs* in the pan-cancer dataset from cBioPortal. **(B–D)** Genetic alterations of *PTBPs* in specific tumor types, in descending order of alteration frequency.

We next used the public dataset “Pan-cancer analysis of whole genomes (ICGC/TCGA, Nature 2020)” from cBioPortal to examine copy number alterations and mutations. The dataset includes a total of 2565 patients, and information on these three genes was available in 174 patients. The copy number alterations and mutation data of *PTBPs* in pan-cancer are shown in Figure 2A. *PTBP1* was the most altered among the *PTBPs*, with the main genetic alteration types being amplifications and deep deletions. *PTBP1* was frequently altered in colorectal cancer, pancreatic cancer, and ovarian cancer; *PTBP2* was frequently altered in non-small cell lung cancer, lung cancer, and ovarian cancer; and *PTBP3* was frequently altered in pancreatic cancer, soft tissue sarcoma, and colorectal cancer (Figures 2B–D).

### *PTBPs* are clinically significant tumor-associated factors

**FIGURE 3:**
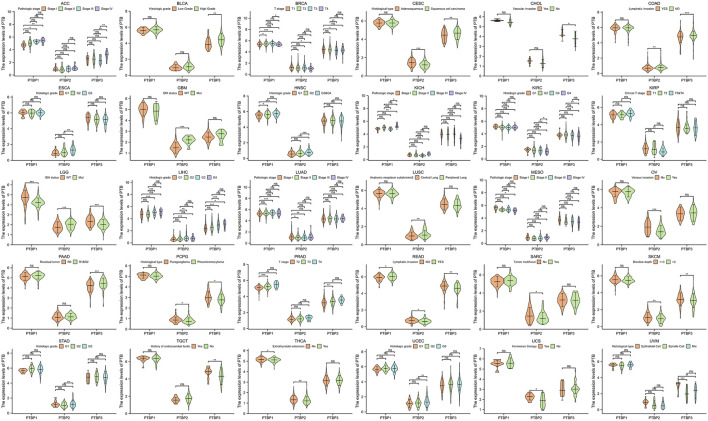
Expression levels of *PTBPs* in clinical parameters of interest (*p < 0.05; **p < 0.01; ***p < 0.001; ns: not significant).

**FIGURE 4:**
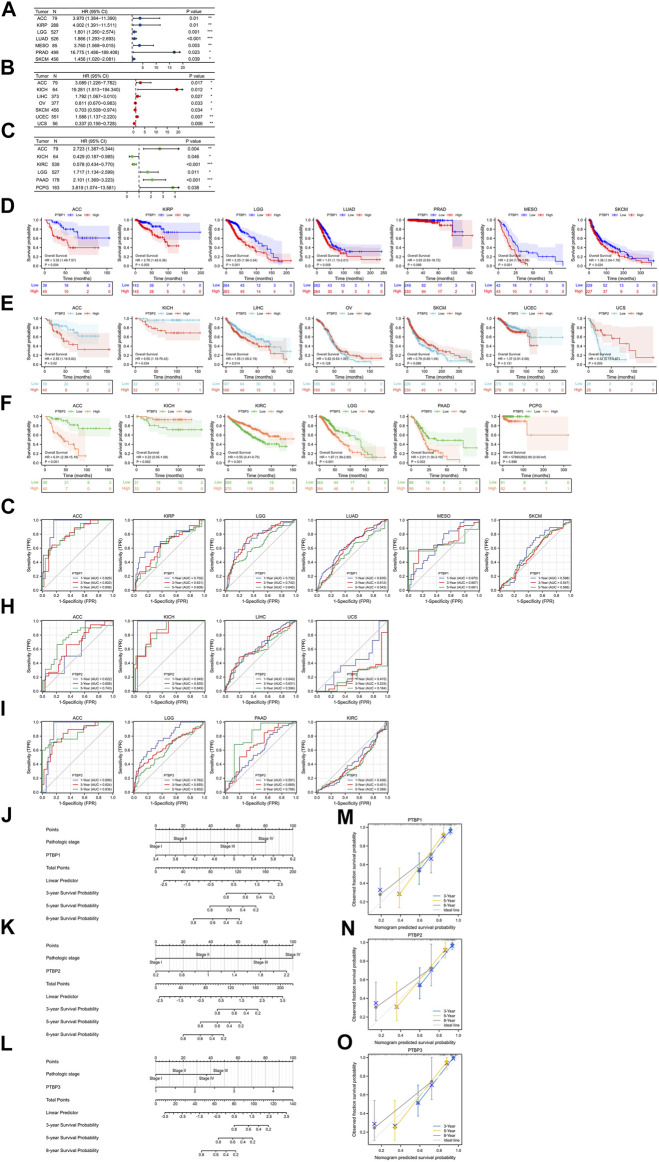
Evaluation of the prognostic value of *PTBPs* in pan-cancer. The multivariate Cox regression analysis results of **(A)** *PTBP1*, **(B)** *PTBP2*, and **(C)** *PTBP3* were visualized and presented as forest plots (*p < 0.05; **p < 0.01; ***p < 0.001). **(D–F)** OS-KM survival curves of *PTBPs* in multiple cancers. **(G–I)** Time-dependent ROC curves of *PTBPs* to evaluate the utility of *PTBPs* as prognostic markers in selected tumor types. **(J–O)** Prognostic nomograms of *PTBP* expression combined with pathological stage in ACC. **(M–O)** The calibration curves for nomograms.

We next analyzed the relationship between *PTBP* expression and stage, grade, or other clinical features in pan-cancer and drew violin plots using data from TCGA (Figure 3). The expression of *PTBP* has a strong correlation with the clinical characteristics of various tumors, indicating that *PTBPs* are related to the occurrence and development of tumors. For example, the expression of *PTBP3* was significantly higher in high-grade BLCA than that in low-grade BLCA (*p* = 0.002). *PTBP2* expression in IDH-mutant GBM was significantly higher than that in IDH-wildtype GBM (*p* < 0.001). The expression of *PTBP1* was related to the pathological stage of ACC: *PTBP1* expression in stage IV ACC was significantly higher than that in stage I ACC (*p* = 0.004). Multivariate Cox regression analysis showed that the HR of *PTBP1* in ACC was 3.97 (*p* = 0.01) (Figure 4A). Patients with high expression of *PTBP1* had a lower probability of survival (Figure 4D), indicating that *PTBP1* is an independent risk factor for ACC and has the potential to be a prognostic indicator.

### Prognostic value of *PTBPs* in pan-cancer

We downloaded RNA-seq data and corresponding clinical information of tumor tissues from 30 cancer types in TCGA database. Univariate and multivariate Cox regression analyses were performed on the OS data to analyze the hazard ratio (HR), 95% confidence interval (95%CI), and *p* value of *PTBPs* in pan-cancer (Table 1). Through multivariate Cox regression analysis, we found that *PTBP1* was significantly associated with poor prognosis in ACC, KIRP, LGG, LUAD, MESO, PRAD, and SKCM (HR > 1, *p* < 0.05). *PTBP2* is a risk factor for ACC, KICH, LIHC, and UCEC (HR > 1, *p* < 0.05) and a protective factor for OV, SKCM, and UCS (HR < 1, *p* < 0.05). *PTBP3* was significantly associated with poor prognosis in ACC, LGG, PAAD, and PCPG (HR > 1, *p* < 0.05), but predicted better prognosis in KICH and KIRC (HR < 1, *p* < 0.05). The above-listed tumors were chosen to make the forest plots (Figures 4A–C).

**TABLE 1:** Univariate and multivariate Cox regression analysis of PTBP expressions and overall survival in pan-cancer.

Cancer (OS)	n	PTBP1 univariate analysis			PTBP1 multivariate analysis			PTBP2 univariate analysis			PTBP2 multivariate analysis			PTBP3 univariate analysis			PTBP3 multivariate analysis		
		HR (95%CI)	*p* value		HR (95%CI)	*p* value		HR (95%CI)	*p* value		HR (95%CI)	*p* value		HR (95%CI)	*p* value		HR (95%CI)	*p* value	
ACC	79	6.748 (2.433–18.713)	<0.001	***	3.970 (1.384–11.390)	0.010	**	4.346 (1.859–10.159)	<0.001	***	3.089 (1.226–7.782)	0.017	*	4.343 (2.376–7.940)	<0.001	***	2.723 (1.387–5.344)	0.004	**
BLCA	413	0.849 (0.551–1.308)	0.457					0.828 (0.554–1.237)	0.357					0.892 (0.711–1.120)	0.325				
BRCA	1082	0.911 (0.532–1.560)	0.733					0.787 (0.529–1.171)	0.238					1.226 (0.924–1.628)	0.158				
CESC	306	1.139 (0.475–2.732)	0.771					0.640 (0.338–1.215)	0.173					1.012 (0.641–1.598)	0.96				
CHOL	36	0.450 (0.066–3.089)	0.416					0.711 (0.269–1.876)	0.491					0.464 (0.163–1.322)	0.151				
COAD	477	1.239 (0.747–2.056)	0.406					0.906 (0.537–1.529)	0.711					0.890 (0.682–1.162)	0.392				
ESCA	162	1.121 (0.496–2.536)	0.783					0.931 (0.514–1.687)	0.814					1.208 (0.729–2.001)	0.463				
GBM	168	1.061 (0.765–1.472)	0.723					0.684 (0.468–1.002)	0.051					1.012 (0.672–1.523)	0.954				
HNSC	501	0.988 (0.670–1.456)	0.950					0.640 (0.406–1.007)	0.054					1.054 (0.823–1.350)	0.675				
KICH	64	38.073 (2.640–549.096)	0.008	**	4.222 (0.280–63.719)	0.298	ns	44.174 (6.571–296.980)	<0.001	***	19.281 (1.913–194.340)	0.012	*	0.425 (0.191–0.945)	0.036	*	0.429 (0.187–0.985)	0.046	*
KIRC	539	1.267 (0.769–2.086)	0.353					1.232 (0.832–1.825)	0.298					0.578 (0.434–0.770)	<0.001	***	0.578 (0.434–0.770)	<0.001	***
KIRP	288	4.002 (1.391–11.511)	0.010	**	4.002 (1.391–11.511)	0.010	**	0.692 (0.342–1.402)	0.306					1.318 (0.837–2.076)	0.233				
LGG	527	2.424 (1.802–3.262)	<0.001	***	1.801 (1.260–2.574)	0.001	***	0.684 (0.442–1.060)	0.089					2.702 (1.900–3.844)	<0.001	***	1.717 (1.134–2.599)	0.011	*
LIHC	373	1.850 (1.256–2.725)	0.002	**	1.663 (0.994–2.782)	0.053	ns	2.087 (1.370–3.179)	<0.001	***	1.792 (1.067–3.010)	0.027	*	1.382 (1.068–1.789)	0.014	*	0.909 (0.617–1.338)	0.627	ns
LUAD	526	1.866 (1.293–2.693)	<0.001	***	1.866 (1.293–2.693)	<0.001	***	0.792 (0.586–1.072)	0.131					1.184 (0.923–1.517)	0.183				
LUSC	496	1.030 (0.728–1.456)	0.868					0.934 (0.666–1.311)	0.695					1.238 (0.944–1.625)	0.123				
MESO	85	3.760 (1.568–9.015)	0.003	**	3.760 (1.568–9.015)	0.003	**	0.964 (0.448–2.072)	0.925					1.377 (0.916–2.070)	0.124				
OV	377	0.948 (0.707–1.272)	0.723					0.811 (0.670–0.983)	0.033	*	0.811 (0.670–0.983)	0.033	*	1.160 (0.922–1.458)	0.205				
PAAD	178	1.082 (0.561–2.087)	0.814					0.600 (0.326–1.106)	0.102					2.101 (1.369–3.223)	<0.001	***	2.101 (1.369–3.223)	<0.001	***
PCPG	183	7.151 (0.269–190.171)	0.240					0.053 (0.001–2.006)	0.113					3.819 (1.074–13.581)	0.038	*	3.819 (1.074–13.581)	0.038	*
PRAD	499	23.975 (2.154–266.880)	0.010	**	16.775 (1.486–189.408)	0.023	*	5.099 (0.489–53.212)	0.173					3.753 (0.941–14.970)	0.061				
READ	166	0.448 (0.162–1.238)	0.122					1.281 (0.287–5.718)	0.745					0.629 (0.335–1.180)	0.149				
SARC	263	2.586 (1.552–4.310)	<0.001	***	3.207 (0.635–16.210)	0.159	ns	0.918 (0.690–1.220)	0.554					1.278 (0.905–1.806)	0.163				
SKCM	456	1.491 (1.048–2.122)	0.026	*	1.456 (1.020–2.081)	0.039	*	0.685 (0.494–0.950)	0.023	*	0.703 (0.508–0.974)	0.034	*	0.892 (0.722–1.103)	0.292				
STAD	370	0.742 (0.532–1.036)	0.079					1.193 (0.838–1.699)	0.326					0.856 (0.663–1.105)	0.232				
THCA	510	1.098 (0.088–13.772)	0.942					2.768 (0.717–10.688)	0.140					1.960 (0.615–6.249)	0.255				
TGCT	139	0.476 (0.031–7.289)	0.594					0.364 (0.036–3.691)	0.393					3.799 (0.587–24.563)	0.161				
UCEC	551	0.716 (0.425–1.206)	0.209					1.588 (1.137–2.220)	0.007	**	1.588 (1.137–2.220)	0.007	**	1.008 (0.777–1.307)	0.955				
UCS	56	1.653 (0.665–4.110)	0.280					0.337 (0.156–0.728)	0.006	**	0.337 (0.156–0.728)	0.006	**	1.335 (0.786–2.268)	0.285				
UVM	80	1.319 (0.341–5.098)	0.688					0.891 (0.356–2.231)	0.805					1.822 (0.967–3.434)	0.064				

We divided samples into high and low expression groups using the median value of *PTBP* expression, predicted survival possibility, and plotted OS-KM curves. Except for PRAD (*p* > 0.05), the survival time of patients with ACC, KIRP, LGG, LUAD, MESO, and SKCM was shorter when *PTBP1* was highly expressed, with statistical significance (HR > 1, *p* ≤ 0.05) (Figure 4D). Patients with ACC, KICH, and LIHC with high *PTBP2* expression had a shorter OS (HR > 1, *p* ≤ 0.05), while patients with UCS with high *PTBP2* expression had a longer survival time (HR = 0.32, *p* = 0.003). No significant differences in OS were observed in OV, SKCM, and UCEC (*p* > 0.05) (Figure 4E). When *PTBP3* was highly expressed, patients with ACC, LGG, and PAAD had lower survival probability and poorer prognosis (HR > 1, *p* ≤ 0.05), while patients with KIRC had a longer OS and better prognosis (HR = 0.55, *p* < 0.001); there was no significant differences observed in patients with KICH and PCPG (*p* > 0.05) (Figure 4F).

By comprehensively analyzing the results of multivariate Cox regression and the OS-KM curve, we concluded that *PTBP1* is a risk factor for ACC, KIRP, LGG, LUAD, MESO, and SKCM, and high expression of *PTBP1* predicts a shorter survival time. *PTBP2* is a risk factor in ACC, KICH, and LIHC and a protective factor in UCS. *PTBP3* is a risk factor for ACC, LGG, and PAAD but a protective factor for KIRC.

Next, we assessed the prognostic value of the three genes in the above tumors. We analyzed the predictive ability of *PTBP* genes for prognosis at 1, 3, and 5 years by time-dependent ROC curves to confirm the accuracy of these candidate markers (Supplementary Table S3). Our results indicated that *PTBP1* may serve as a prognostic biomarker for ACC, KIRP, and LGG at the three time points according to the criterion of AUC >0.7 (Figure 4G). *PTBP2* showed good prognostic value in ACC and KICH (Figure 4H), and *PTBP3* showed good prognostic value in ACC, LGG, and PAAD (Figure 4I), indicating these *PTBPs* may function as prognostic biomarkers in these tumors.

Our results showed that all three *PTBPs* were associated with poor prognosis in ACC, and therefore we determined the prognostic nomogram of ACC. Pathologic stage and *PTBP* expression were included in Cox regression analysis to establish prognostic nomograms. A vertical line was drawn to connect corresponding points and calculate the total score to estimate the 3-, 5-, and 8-years survival probability of ACC patients (Figures 4J–L). Calibration curves used to observe the predictive effect of the nomogram are shown in Figures 4M–O.

### The expression of *PTBPs* in tumor immune subtypes

In 2018, Scientists performed an extensive immune genomic analysis of 33 cancer types (Thorsson et al., 2018). Six immune subtypes, including C1 (wound healing), C2 (IFN-gamma dominant), C3 (inflammatory), C4 (lymphocyte depleted), C5 (immunologically quiet), and C6 (TGF-β dominant), were identified by macrophage and lymphocyte markers, the ratio of Th1 cells to Th2 cells, and immune regulatory genes. This tumor heterogeneity leads to suboptimal outcomes of immunotherapy in the clinic.

**FIGURE 5:**
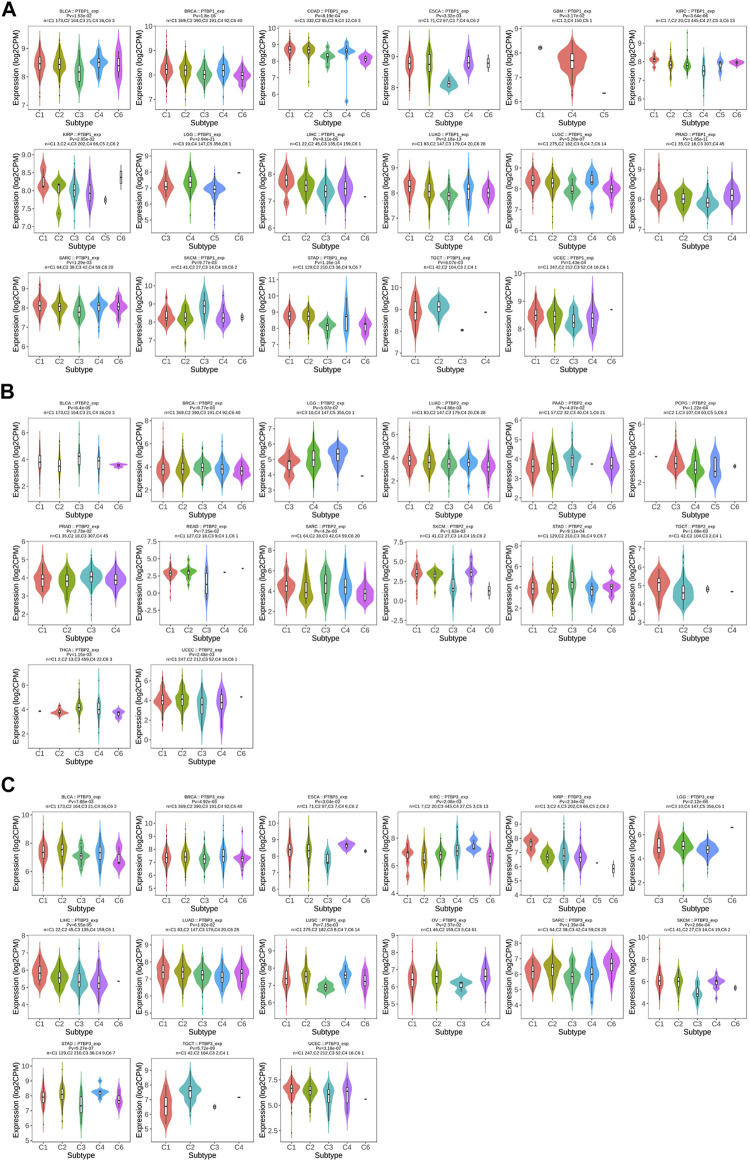
The relationship between *PTBP* expression and tumor immune subtype. **(A)** Expression levels of *PTBP1* in immune subtypes. **(B)** Expression levels of *PTBP2* in immune subtypes. **(C)** Expression levels of *PTBP3* in immune subtypes. These results are statistically significant.

We investigated the expression levels of *PTBPs* in different tumor immune subtypes by TISIDB. The results showed that *PTBP1* expression was associated with tumor immune subtypes of BLCA, BRCA, COAD, ESCA, GBM, KIRC, KIRP, LGG, LIHC, LUAD, LUSC, PRAD, SARC, SKCM, STAD, TGCT, and UCEC (Figure 5A). *PTBP2* expression correlated with tumor immune subtypes of BLCA, BRCA, LGG, LUAD, PAAD, PCPG, PRAD, READ, SARC, SKCM, STAD, TGCT, THCA, and UCEC (Figure 5B). *PTBP3* expression correlated with tumor immune subtypes of BLCA, BRCA, ESCA, KIRC, KIRP, LGG, LIHC, LUAD, LUSC, OV, SARC, SKCM, STAD, TGCT, and UCEC (Figure 5C). The expression of *PTBPs* in immune subtypes of other cancers is shown in Supplementary Figures S5–S7.

### *PTBP* expression and immune infiltrating cells in the tumor microenvironment

**FIGURE 6:**
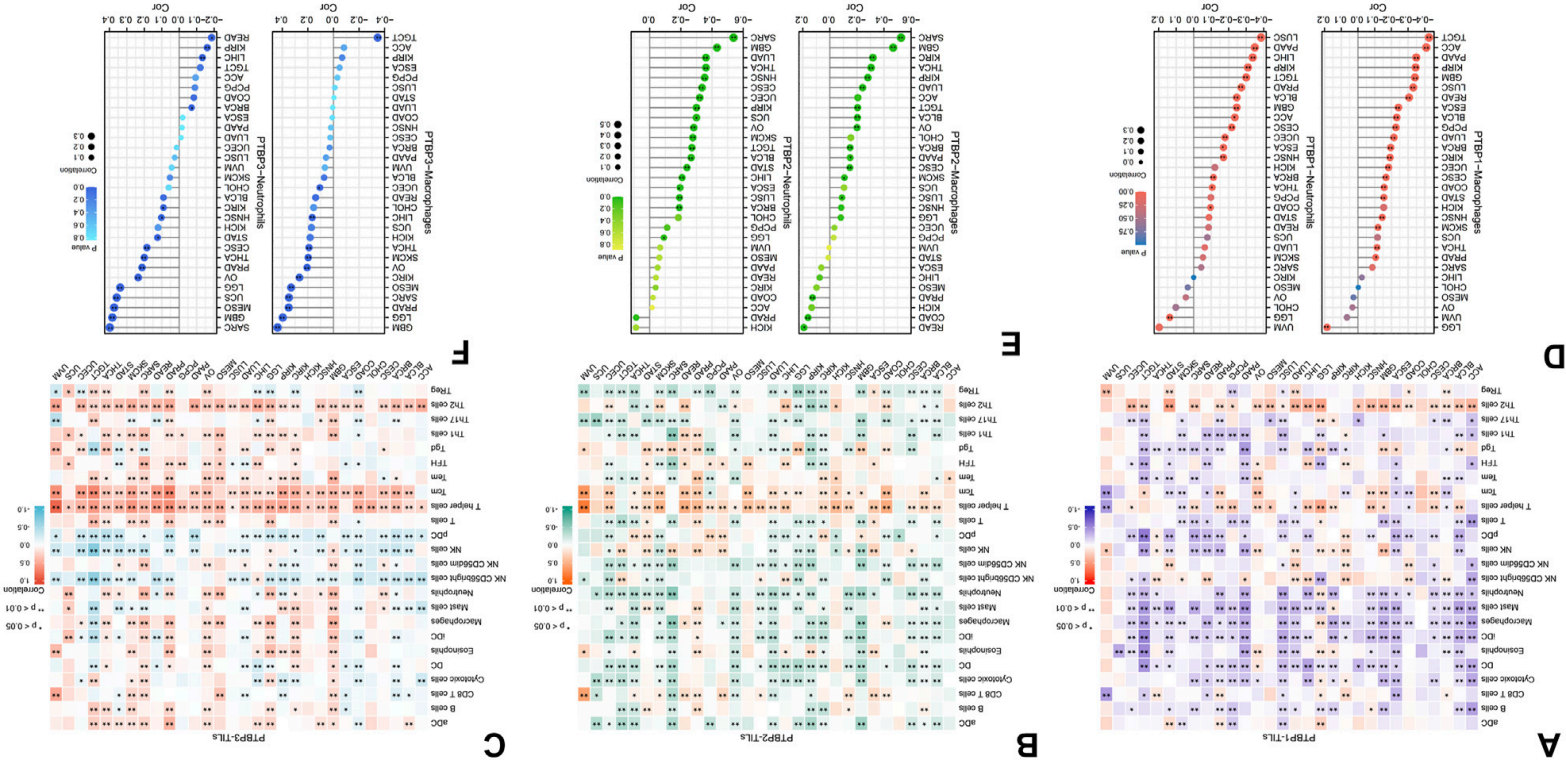
Correlation of *PTBP* expression with tumor-infiltrating lymphocytes in the tumor microenvironment in pan-cancer. **(A–C)** Heatmap of the correlation of *PTBP* expression with 24 TILs in pan-cancer. **(D–F)** The correlation of *PTBP* expression with macrophages and neutrophils in various tumors is shown in detail in lollipop plots (*p < 0.05; **p < 0.01).

The Spearman correlations between *PTBPs* and TILs in various tumor types were further investigated. We found strong positive correlations of *PTBP1* with Th2 cells (Figure 6A), *PTBP2* with T helper cells and Tcm (Figure 6B), and *PTBP3* with T helper cells, Tcm, and Th2 cells (Figure 6C) in most tumor types. Overall, the expression of *PTBPs* was highly correlated with the number of TILs in the TME. These results suggest that PTBPs may have a regulatory effect on the tumor microenvironment (TME) (Geng et al., 2021).

We also examined *PTBP* expression with macrophages and neutrophils (Figures 6D–F). Most tumor types had an infiltration of macrophages and neutrophils, and this was inversely linked with the expression of *PTBP1* and *PTBP2*. However, in many tumor types, *PTBP3* levels were favorably linked with macrophage and neutrophil counts. These details are shown in Supplementary Table S4.

### Correlation analysis of *PTBPs* and immune checkpoint

**FIGURE 7:**
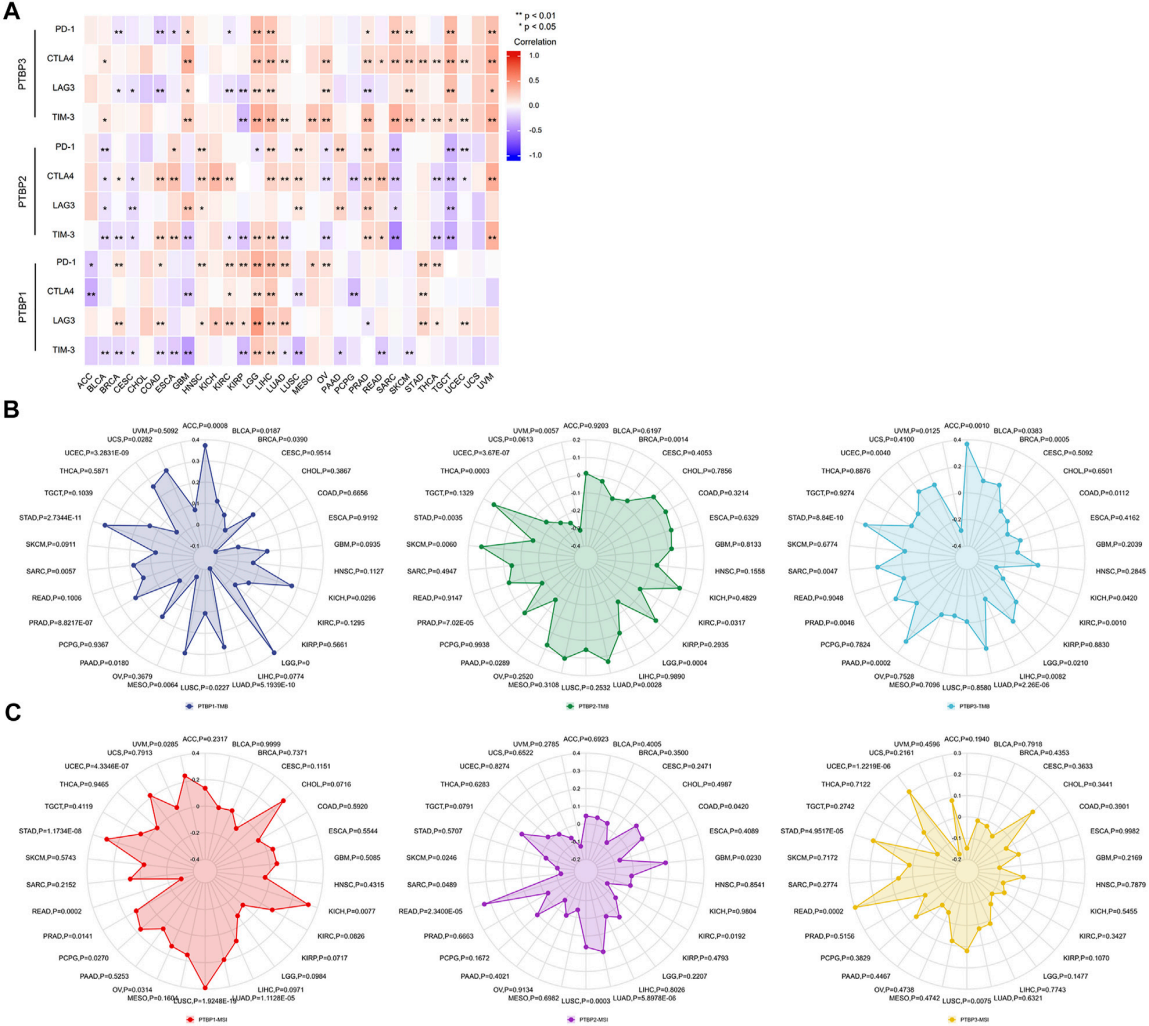
Spearman correlation analysis of *PTBP* expression with immune checkpoint genes, tumor mutational burden and microsatellite instability in pan-cancer. **(A)** Correlation of *PTBP* expression with ICPs (PD-1, CTLA4, LAG3 and TIM-3) in pan-cancer (*p < 0.05; **p < 0.01). **(B)** Correlation of *PTBP* expression with TMB. **(C)** Correlation of *PTBP* expression with MSI. *p* values are marked in the figures.

Studies have shown that the immune checkpoint (ICP) genes have a great influence on the efficacy of immunotherapy. PD-1, CTLA4, LAG3, and TIM-3 are four ICPs that are frequently examined in the clinic, and inhibitors targeting these factors have shown potent tumor-killing effects in a variety of tumors (Sun et al., 2021; Yang et al., 2021; Gaikwad et al., 2022; Tian et al., 2022). To explore the potential of *PTBPs* in immunotherapy, we analyzed the relationship between *PTBPs* and four ICP genes in pan-cancer (Supplementary Table S5). In the 30 tumor types, *PTBP1* expression had the most prevalent positive correlation with *LAG3* and *PD-1* expression (in Figure 7A). Similarly, *PTBP2* was generally positively correlated with *CTLA4* and PTBP3 with *CTLA4* and *TIM-3*. These results suggest that *PTBPs* may serve as potential targets for immunotherapy.

### *PTBP* expression correlates with tumor mutational burden and microsatellite instability and can predict immunotherapy efficacy

TMB and MSI are demonstrated biomarkers that predict the efficacy of immunotherapy, with higher TMB or MSI indicating a better response to ICP inhibitors (Chan et al., 2019; Diao et al., 2021). Using the criteria of |R| ≥ 0.3 and *p* < 0.05, the radar chart showed that the expression of *PTBP1* in ACC, LGG, MESO, and STAD was positively correlated with TMB. *PTBP2* expression was negatively correlated with TMB in UVM. *PTBP3* expression was positively correlated with TMB in ACC and STAD (Figure 7B).

In KICH and LUSC, *PTBP1* expression associated favorably with MSI, but it correlated negatively in READ. *PTBP2* expression was positively correlated with MSI in READ (Figure 7C). The detailed expression data were presented in Supplementary Table S6, and the correlations were shown in Supplementary Table S7. These evidences supported the finding that *PTBPs* may predict response to immunotherapy and play a role in tumor immunity.

### Correlation analysis of *PTBPs* and chemical drug sensitivity

**FIGURE 8:**
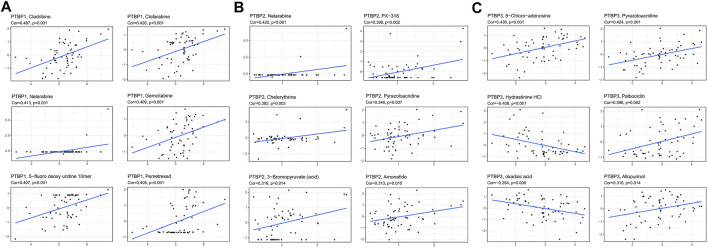
Pearson correlation of *PTBP* expression with drug sensitivity scores in various tumor cell lines in Cellminer. The top six drugs with the largest absolute value of the correlation coefficient are displayed. **(A)** Correlation of *PTBP1* with drug sensitivity. **(B)** Correlation of *PTBP2* with drug sensitivity. **(C)** Correlation of *PTBP3* with drug sensitivity. The correlation coefficient and *p* value are marked in the figure.

We next analyzed the Pearson correlation of *PTBP* expression with the sensitivity of 263 FDA-approved drugs in 60 tumor cell lines using the Cellminer database (Figures 8A–C) and obtained the top six drugs with the strongest correlation with *PTBPs*. For example, the expression of *PTBP1* was proportional to the sensitivity of cells to gemcitabine (R = 0.409, *p* = 0.001): the higher the expression of *PTBP1*, the more sensitive the cell was to gemcitabine. Therefore, the expression of *PTBPs* may be a predictor of tumor response to chemotherapeutic drugs.

### The analysis of protein-protein interaction

**FIGURE 9:**
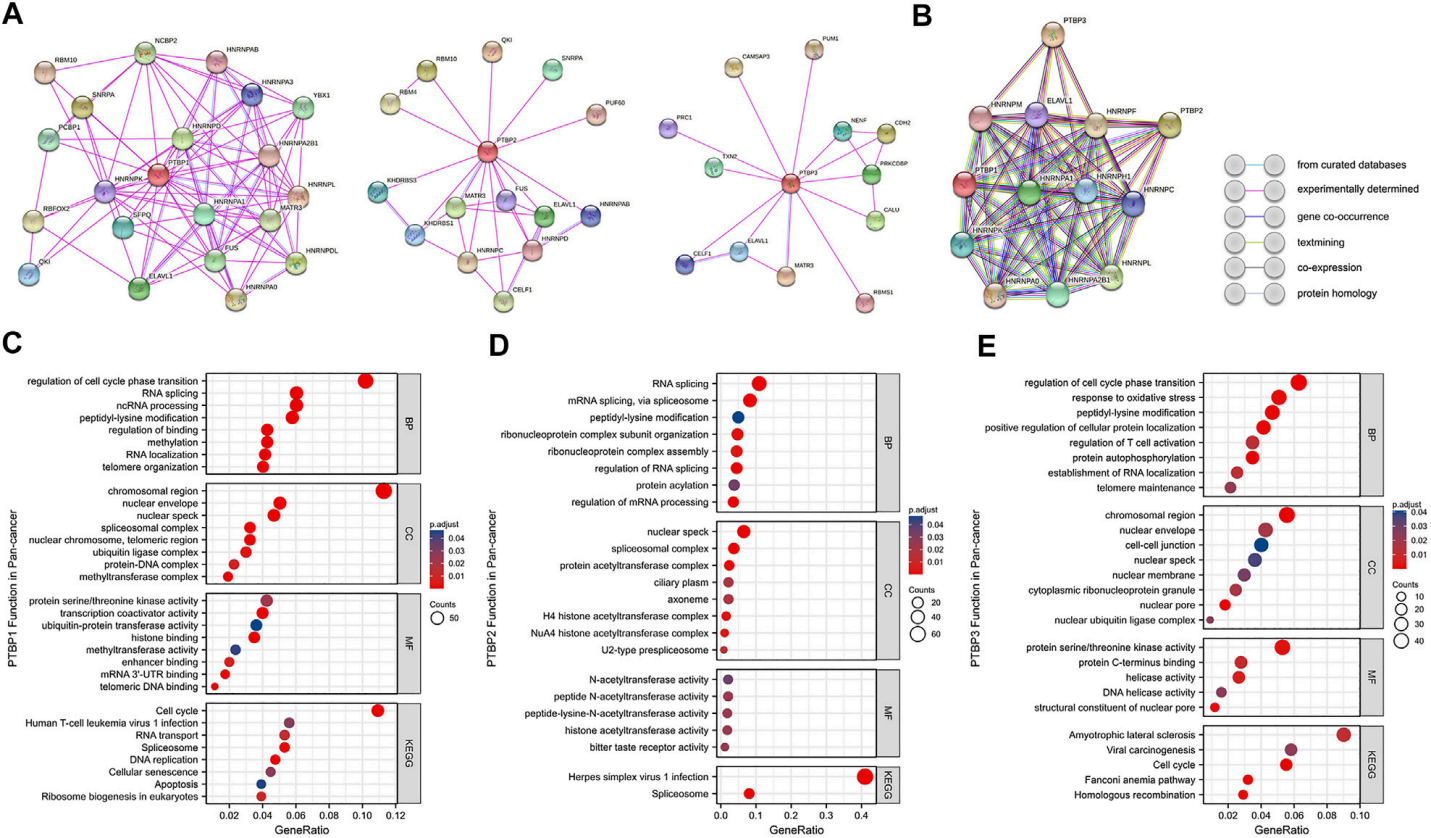
Protein-protein interaction networks and functional enrichment analysis of PTBPs in pan-cancer. **(A)** Experimentally validated interacting proteins of PTBPs using STRING. **(B)** The interaction relationship among the three protein molecules. Line colors in the legend indicate different relationships. **(C–E)** GO enrichment and KEGG pathway analysis results for co-expressed mRNAs of *PTBPs*.

We mapped the PPI networks of PTBP1, PTBP2, and PTBP3 (Figure 9A) respectively and visualized the interaction among these three molecules using STRING (Figure 9B). It showed that PTBP1 was closely related to heterogeneous nuclear ribonucleoproteins (hnRNPs), YBX1, and SFPQ (Meissner et al., 2000; King et al., 2014). There are four relationships between PTBP1 and PTBP2: experimentally determined interactions, databases recorded interactions, protein homology, and text mining. However, PTBP3 did not seem to interact with PTBP1 and PTBP2 (Figure 9B).

### Functional enrichment analysis of *PTBP*-related genes

Spearman correlation analysis on the pan-cancer dataset from cBioPortal yielded 926 mRNAs co-expressed with PTBP1, 657 mRNAs co-expressed with PTBP2, and 874 mRNAs co-expressed with PTBP3 (|R| ≥ 0.4, *p* < 0.05) (Supplementary Table S8). We further analyzed *PTBP*-related mRNAs using GO (including BP, CC, and MF) and KEGG enrichment analyses (Figures 9C–E). The results revealed that PTBP1 may function through “Cell cycle,” “Human T-cell leukemia virus one infection,” “RNA transport,” “Spliceosome,” “DNA replication,” “Cellular senescence” and “Apoptosis.” PTBP2 may be associated with “Herpes simplex virus one infection” and “Spliceosome” pathways, while PTBP3 may affect tumor progression through “Amyotrophic lateral sclerosis,” “Viral carcinogenesis,” “Cell cycle” and “Homologous recombination” pathways (Supplementary Table S9). Together, the results above have established a novel theoretical framework for the investigation of PTBP regulation mechanism in malignancies.

## Discussion

PTBPs are RNA-binding proteins that are involved in alternative splicing, mRNA stability, and translation. The PTBP family includes PTBP1, PTBP2 and PTBP3. PTBP1 can be expressed in almost all types of cells; PTBP2 is only expressed in the neurological system while PTBP3 is found mostly in immune cells (Spellman et al., 2007). Among the PTBP family members, PTBP1 is most frequently linked with cancer, followed by PTBP3. It is reported that PTBP1 promotes lung cancer metastasis by regulating the alternative splicing of *Mena* mRNA (Li et al., 2019). PTBP3 is upregulated in breast cancer and regulates *ZEB1* mRNA stability to promote epithelial-mesenchymal transition in BRCA (Hou et al., 2018; Liang et al., 2020). Most research has focused on the function of PTBPs in tumor cells, but little attention has been paid to their interaction with immune cells in the TME (Sasanuma et al., 2019; Geng et al., 2021). In addition, reports of PTBPs in uncommon tumors are rare. Thus, we investigated the expression, function, and immune characterization of PTBP1, PTBP2, and PTBP3 in pan-cancer.

We first performed differentiation analysis and correlation analysis on the expression of the *PTBP1*, *PTBP2*, and *PTBP3* genes in 30 tumor types using TCGA. The results showed that in most tumor types, the expression levels of *PTBP1* and *PTBP3* in tumor tissues were significantly higher than that in non-tumor tissues. In contrast, the expression level of *PTBP2* was lower in tumor tissues compared with that in normal tissues. Interestingly, when we analyzed the expression correlation of *PTBPs*, we found that *PTBP1/3* appeared to have opposite expression trends to *PTBP2* in pan-cancer. Our results were consistent with other scholars’ findings. Previous studies suggested that PTBP1 is a repressor of PTBP2 and that there was a “switch” between the two molecules (Boutz et al., 2007; Spellman et al., 2007). SON may be an on-off regulator of the expression of *PTBP1* and *PTBP2* in GBM (Kim et al., 2021), and PTBP2 compensates for the absence of *Ptbp1* during B cell development in mice (Monzón-Casanova et al., 2020). Furthermore, we found a co-expression trend between *PTBP1* and *PTBP3* which deserved to be further investigated.

It has been reported in the literature that PTBP1 can be used as a biomarker for poor prognosis in bladder cancer (Bielli et al., 2018), and PTBP3 as a therapeutic target for gastric cancer (Liang et al., 2017). Here we further comprehensively explored the association of *PTBP* expression with prognosis in pan-cancer. Through multivariate Cox regression analysis and OS-KM survival curves, we found that patients with ACC, LGG, and PAAD had poor prognosis when *PTBP3* was highly expressed, but patients with KIRC had better prognosis. Given that the expression of *PTBP3* in KIRC tumor tissues was significantly lower than that in control tissues, PTBP3 may be a tumor suppressor molecule in KIRC. Thus, more research is required to examine the function and molecular mechanism of PTBP3 in KIRC. Time-dependent ROC curves were used to verify the prognostic value of *PTBPs* in pan-cancer. Compared with the ordinary ROC curve, the time-dependent ROC curve detects the accuracy of candidate markers at specified times. We finally identified *PTBP1* in ACC, KIRP, and LGG; *PTBP2* in ACC and KICH; and *PTBP3* in ACC, LGG, and PAAD as potential prognostic biomarkers that may be involved in tumor progression in these tumor types.

We then analyzed the expression of *PTBPs* in different immune subtypes. The results indicated that *PTBPs* might participate in immune regulation. The expression of *PTBPs* was significantly different across multiple immune subtypes and strongly correlated with the number of TILs in the TME. Remarkably, *PTBP1* on Th2 cells, *PTBP2* on T helper cells and Tcm, and *PTBP3* on T helper cells, Tcm, and Th2 cells may have broad positive regulatory effects in pan-cancer. *PTBPs* are also strongly associated with macrophages and neutrophils in the TME. For example, *PTBP3* expression was positively correlated with macrophages and neutrophils in GBM, LGG, PRAD, SARC, MESO, KIRC, OV, and THCA. These results demonstrated the important role of PTBPs in tumor immunity and the tumor microenvironment.

The immune microenvironment in tumor tissues leads to tumor heterogeneity, which influences the clinical efficacy of anticancer drugs. Immune checkpoint inhibitors are used as treatment options for cancer patients. We found that *PTBP* expression showed a strong correlation with *PD-1*, *CTLA4*, *LAG3*, or *TIM-3* in pan-cancer. Therefore, *PTBPs* may be a class of potential therapeutic targets, providing a new direction for combined targeted immunotherapy in the future.

We also analyzed the correlation of *PTBPs* with TMB and MSI. Tumor cells with high TMB usually have higher levels of neoantigens, which help the immune system to recognize the tumor and activate the anti-tumor effect of T cells. Therefore, higher TMB generally indicates better outcome of immunotherapy, and TMB is highly correlated with the efficacy of PD-1/PD-L1 inhibitors (Yarchoan et al., 2017; Chan et al., 2019). MSI works similarly. TMB and MSI have become predictive markers of tumor immunotherapy efficacy in recent years. The correlation of *PTBP* expression with TMB and MSI in pan-cancer suggests that PTBPs may become novel biomarkers for predicting patients’ response to immunotherapy.

We also made other notable findings. Gemcitabine is an effective anti-tumor drug for NSCLC (stage III and IV), OV, BRCA, BLCA, and other malignant tumors (Ferrazzi and Stievano, 2006; Mornex and Girard, 2006), and 5-fluorodeoxyuridine is a common chemotherapeutic drug for BRCA, STAD, READ, and BLCA (Koizumi et al., 1993). *PTBP1* expression was proportional to the sensitivity of cells to gemcitabine (R = 0.409, *p* = 0.001) and 5-fluorodeoxyuridine (R = 0.407, *p* = 0.001). This result indicates that the expression of *PTBPs* may predict the therapeutic effect of chemotherapeutic drugs.

When we investigated “interacting proteins”, we found that all PTBP family proteins can tightly interacts with hnRNPs and ELAVL1 (also known as HuR), which was verified by co-immunoprecipitation or reported in literature (Hegele et al., 2012). The presence of such protein complexes may increase their effect. For example, PTBP1 can interact with HuR and jointly upregulate the translation of *HIF-1α* mRNA in human cervical carcinoma HeLa cells (Galbán et al., 2008).

In the enrichment analysis of co-expressed genes, we inferred that PTBPs may function in the cell cycle, RNA splicing and RNA localization. PTBP1 and PTBP3 were enriched in telomere-related signaling pathways, suggesting that they may be involved in cellular senescence pathways. Scientists found that PTBP1 can regulate alternative splicing of genes involved in intracellular trafficking to control the senescence-associated secretory phenotype (SASP). Inhibition of PTBP1 blocks the tumor-promoting effect of SASP and impair immune surveillance (Georgilis et al., 2018). Sayed et al. also found that knockdown of *PTBP1* in cancer cells reduced *hTERT* full-length splicing and telomerase activity (Sayed et al., 2019). The important role of PTBP1 and PTBP3 in cellular senescence and immunity should be further explored.

This study has several limitations. First, our conclusions are limited by sequencing technologies and analytical methodologies from the database, and the data may be lacking in granularity and precision. This has become a pervasive problem in bioinformatics research. Second, whether *PTBPs* can be used as biomarkers for prognosis and immunotherapy requires validation in more clinical samples. At present, there is no immune-targeted drug against PTBPs, so it is not possible to clinically verify the effect of these targets. Third, the involvement of PTBPs in immune regulation and cellular senescence need to be supported by *in vitro* and *in vivo* experimental evidence.

## Conclusion

This study comprehensively and systematically analyzed the prognostic value, genetic variation, and signaling pathways of PTBP1, PTBP2, and PTBP3 and the correlation of *PTBP* expression with TILs, ICP, TMB, MSI, and drug sensitivity from a pan-cancer perspective. Our results indicate that *PTBPs* may be promising prognostic biomarkers and predict the response to immunotherapy in pan-cancer. We found that PTBPs are closely related to tumor progression and cell senescence, which provides a theoretical reference for subsequent research.

## Data Availability

The original contributions presented in the study are included in the article/Supplementary Material, further inquiries can be directed to the corresponding authors.
