# Supplementary material for: PTBPs: An immunomodulatory-related prognostic biomarker in pan-cancer
Source: Front Mol Biosci. 2022 Aug 23;9:968458. doi: 10.3389/fmolb.2022.968458 (PMC9531344; doi:10.3389/fmolb.2022.968458)
Supplement: Supplementary file 3 [file Table1.docx]

**Table S1 Abbreviations for cancer in TCGA**

| **Cancer type** | **Abbreviations** |
| --- | --- |
| adrenocortical carcinoma | ACC |
| bladder urothelial carcinoma | BLCA |
| breast invasive carcinoma | BRCA |
| cervical squamous cell carcinoma | CESC |
| cholangiocarcinoma | CHOL |
| colon adenocarcinoma | COAD |
| esophageal carcinoma | ESCA |
| glioblastoma | GBM |
| brain lower grade glioma | LGG |
| head and neck squamous cell carcinoma | HNSC |
| kidney chromophobe | KICH |
| kidney renal clear cell carcinoma | KIRC |
| kidney renal papillary cell carcinoma | KIRP |
| liver hepatocellular carcinoma | LIHC |
| lung adenocarcinoma | LUAD |
| lung squamous cell carcinoma | LUSC |
| mesothelioma | MESO |
| ovarian serous cystadenocarcinoma | OV |
| pancreatic adenocarcinoma | PAAD |
| pheochromocytoma and paraganglioma | PCPG |
| prostate adenocarcinoma | PRAD |
| rectum adenocarcinoma | READ |
| sarcoma | SARC |
| skin cutaneous melanoma | SKCM |
| stomach adenocarcinoma | STAD |
| testicular germ cell tumors | TGCT |
| thyroid carcinoma | THCA |
| uterine corpus endometrial carcinoma | UCEC |
| uterine carcinosarcoma | UCS |
| uveal melanoma | UVM |
