# Supplementary material for: PTBPs: An immunomodulatory-related prognostic biomarker in pan-cancer
Source: Front Mol Biosci. 2022 Aug 23;9:968458. doi: 10.3389/fmolb.2022.968458 (PMC9531344; doi:10.3389/fmolb.2022.968458)
Supplement: Supplementary file 12 [file DataSheet1.docx]

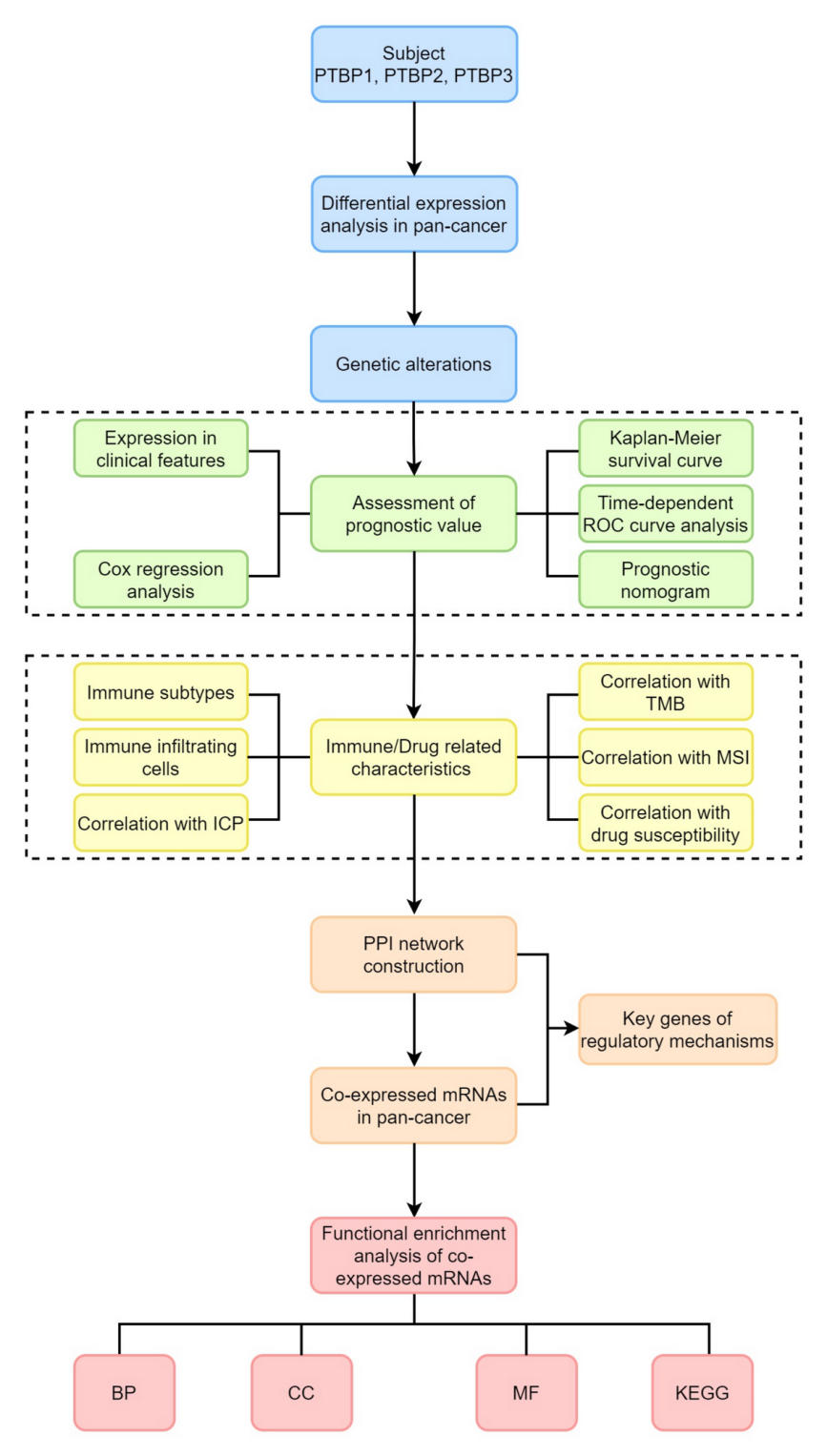


**Supplementary Figure 1.** A flowchart of the study design and analytic approach.


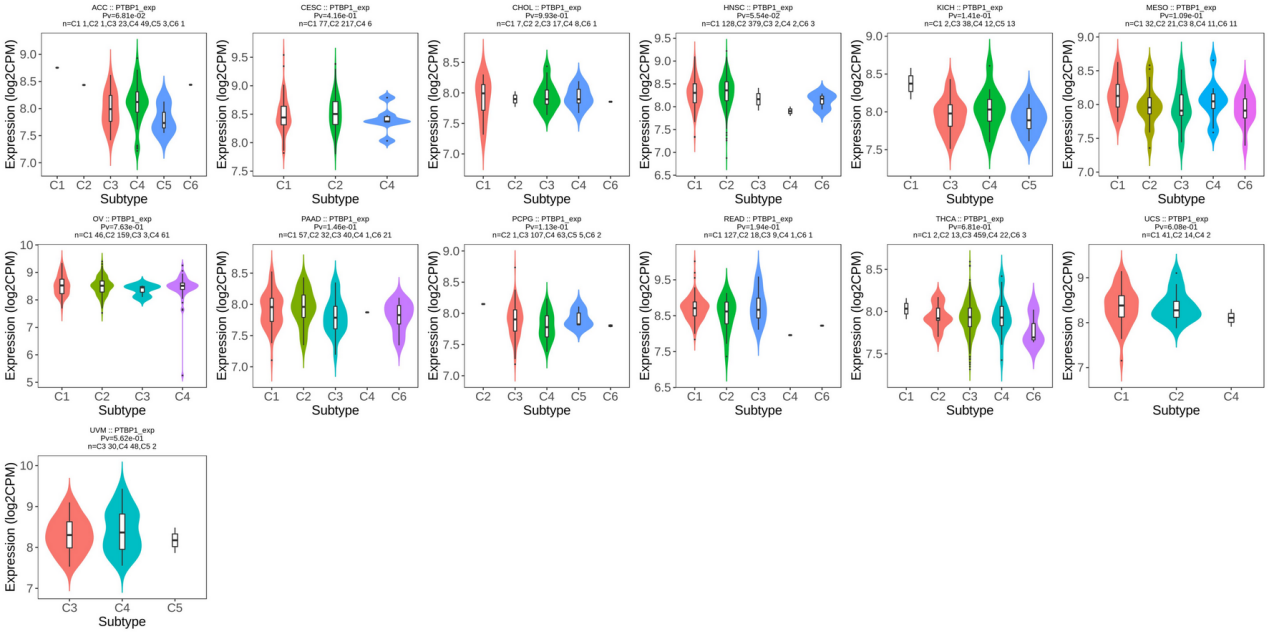


**Supplementary Figure 2.** The relationship between PTBP1 expression and immune subtypes in various tumor types (P > 0.05).


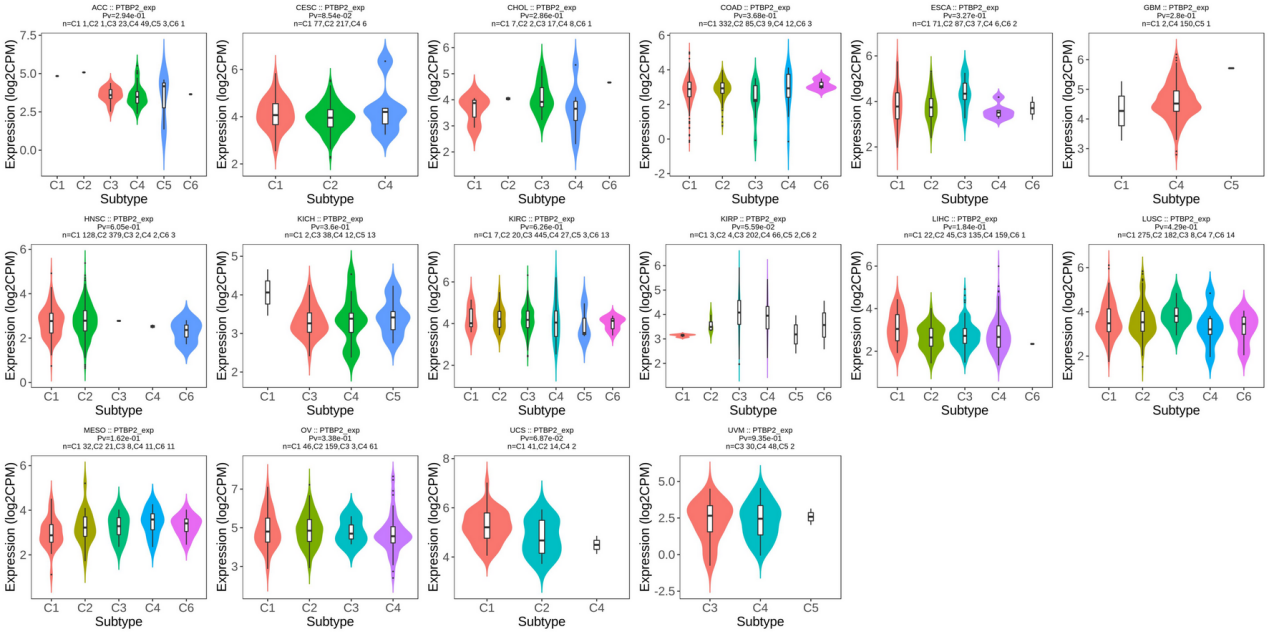


**Supplementary Figure 3.** The relationship between PTBP2 expression and immune subtypes in various tumor types (P > 0.05).


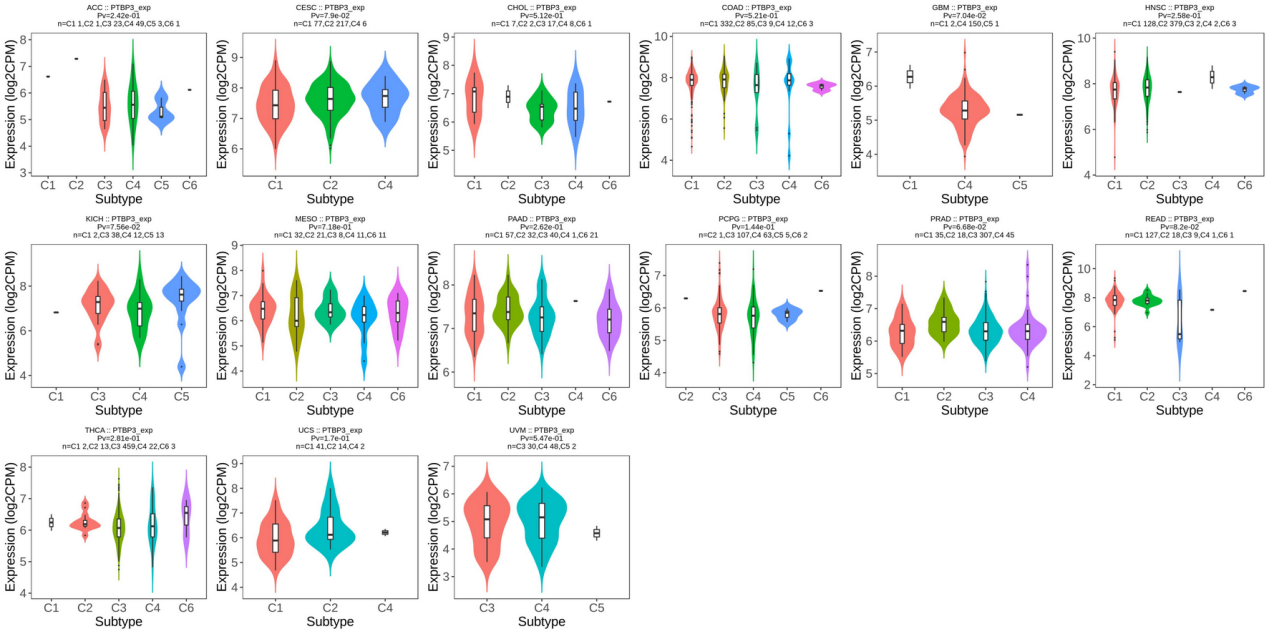


**Supplementary Figure 4.** The relationship between PTBP3 expression and immune subtypes in various tumor types (P > 0.05).

**Supplementary Table 1.** Tumor types and abbreviations in TCGA database.

**Supplementary Table 2.** Expression correlations of the *PTBP* genes in 21 tumor types.

**Supplementary Table 3.** Sensitivity, specificity and cut-off value of *PTBPs* in time-dependent ROC.

**Supplementary Table 4.** Correlations between *PTBPs* and 24 tumor-infiltrating lymphocytes (TILs) in pan-cancer.

**Supplementary Table 5.** Correlations between *PTBPs* and immune checkpoint (ICP) genes in pan-cancer.

**Supplementary Table 6.** *PTBPs* expression, tumor mutational burden (TMB) scores, and microsatellite instability (MSI) scores in pan-cancer.

**Supplementary Table 7.** Correlation of *PTBP* expression with TMB and MSI in pan-cancer.

**Supplementary Table 8.** Genes co-expressed with *PTBPs* analyzed by the cBioPortal database in pan-cancer.

**Supplementary Table 9.** Correlation of *PTBP* expression with target gene expression in various tumor types.

**Supplementary Table 10.** GO enrichment and KEGG pathway analyses of *PTBP*-related mRNAs.
